# Examining Real-World Medication Consultations and Drug-Herb Interactions: ChatGPT Performance Evaluation

**DOI:** 10.2196/48433

**Published:** 2023-08-21

**Authors:** Hsing-Yu Hsu, Kai-Cheng Hsu, Shih-Yen Hou, Ching-Lung Wu, Yow-Wen Hsieh, Yih-Dih Cheng

**Affiliations:** 1 Department of Pharmacy China Medical University Hospital Taichung Taiwan; 2 Graduate Institute of Clinical Pharmacy College of Medicine National Taiwan University Taipei Taiwan; 3 Artificial Intelligence Center China Medical University Hospital Taichung Taiwan; 4 Department of Medicine China Medical University Taichung Taiwan; 5 School of Pharmacy College of Pharmacy China Medical University Taichung Taiwan

**Keywords:** ChatGPT, large language model, natural language processing, real-world medication consultation questions, NLP, drug-herb interactions, pharmacist, LLM, language models, chat generative pre-trained transformer

## Abstract

**Background:**

Since OpenAI released ChatGPT, with its strong capability in handling natural tasks and its user-friendly interface, it has garnered significant attention.

**Objective:**

A prospective analysis is required to evaluate the accuracy and appropriateness of medication consultation responses generated by ChatGPT.

**Methods:**

A prospective cross-sectional study was conducted by the pharmacy department of a medical center in Taiwan. The test data set comprised retrospective medication consultation questions collected from February 1, 2023, to February 28, 2023, along with common questions about drug-herb interactions. Two distinct sets of questions were tested: real-world medication consultation questions and common questions about interactions between traditional Chinese and Western medicines. We used the conventional double-review mechanism. The appropriateness of each response from ChatGPT was assessed by 2 experienced pharmacists. In the event of a discrepancy between the assessments, a third pharmacist stepped in to make the final decision.

**Results:**

Of 293 real-world medication consultation questions, a random selection of 80 was used to evaluate ChatGPT’s performance. ChatGPT exhibited a higher appropriateness rate in responding to public medication consultation questions compared to those asked by health care providers in a hospital setting (31/51, 61% vs 20/51, 39%; *P*=.01).

**Conclusions:**

The findings from this study suggest that ChatGPT could potentially be used for answering basic medication consultation questions. Our analysis of the erroneous information allowed us to identify potential medical risks associated with certain questions; this problem deserves our close attention.

## Introduction

With its impressive ability to perform natural language tasks and its user-friendly interface, ChatGPT has garnered significant attention since its release by OpenAI. ChatGPT is an extension of a generative pretrained transformer (GPT) natural language processing (NLP) model called GPT-3 developed by OpenAI; it represents an advanced iteration known as GPT-3.5. In addition to achieving human-level performance in entertainment-oriented conversations and writing tasks, ChatGPT can also provide satisfactory answers to questions involving many different professional knowledge domains. The field of NLP is experiencing rapid progress, largely due to extensive data from the Internet and computational power advancements in accordance with Moore’s law, and many language models with an even larger size than GPT-3 have been trained, released, and made publicly available [1]. However, before the release of ChatGPT, one needed to fine-tune the models or write carefully engineered text prompts to coax them to do specific tasks, requiring some professional knowledge and effort. Now with ChatGPT, people can easily ask this model to do any kind of natural language task in a conversational way, without writing programming language or carefully engineered text prompts.

Many studies have designed specialized testing procedures to evaluate ChatGPT’s abilities and limitations. In medicine, it can achieve a nearly passing score of 60% accuracy on the US Medical Licensing Exam (USMLE) [2,3]. In programming, its performance in answering questions in an interview test is similar to “level 3” Google engineers [4]. These studies show that this tool has great application potential and may bring disruptive revolutions to the way people work in many fields. At present, there is no specific evaluation of ChatGPT in pharmacy-related work in academia. To better understand ChatGPT’s abilities in this domain, we designed relevant experiments for the pharmaceutical field and evaluated ChatGPT’s ability in the field of pharmacy for public reference.

This exploratory study aimed to understand better the suitability of ChatGPT for answering real-world medication consultation questions in pharmaceutical services. Additionally, we conducted an in-depth analysis of the accuracy of responses to drug-herb interaction questions to assess the potential of ChatGPT in medication education and consultation.

## Methods

### Study Design

**Figure 1:**
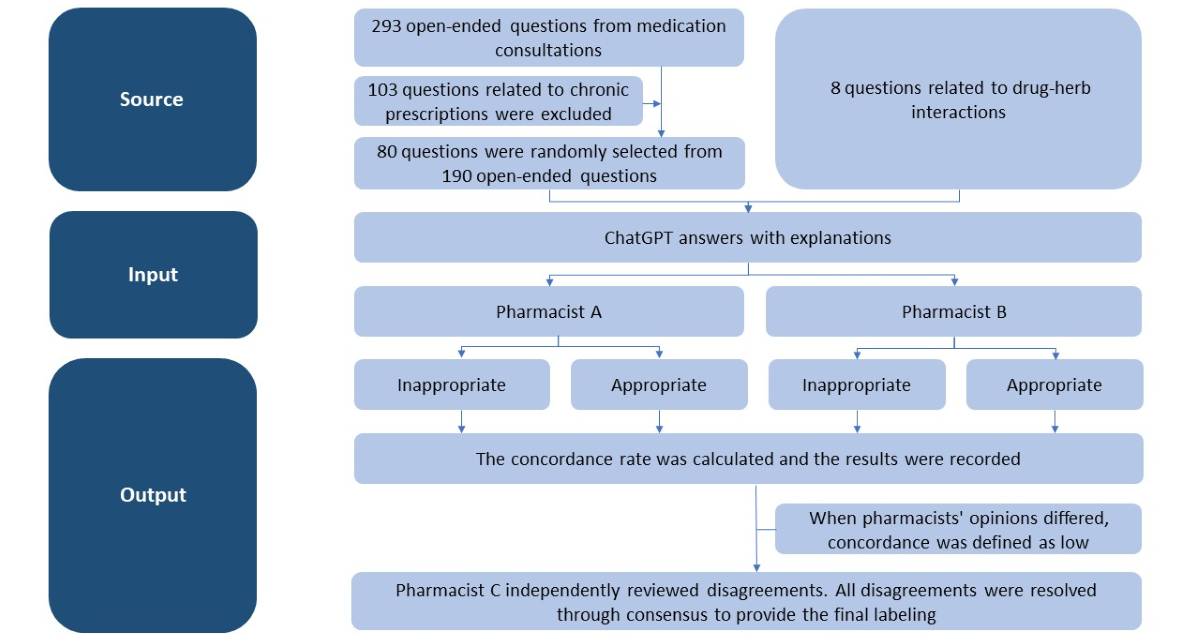
Flowchart of data collection, ChatGPT input, ChatGPT output, and records.

We followed the STROBE (Strengthening the Reporting of Observational Studies in Epidemiology) reporting guidelines for observational studies [5]. Figure 1 shows the study protocol.

Our test questions were divided into 2 groups. The first included 293 open-ended queries; we excluded 103 questions related to chronic prescriptions and medication reservations, then randomly selected 80 of the remaining questions. The second group of questions comprised 8 queries concerning drug interactions between traditional Chinese and Western medicines. All of the questions we evaluated and the responses generated by ChatGPT were in Chinese.

### Language Model

ChatGPT is a large language model that can interact with users in a conversational way. One of its most remarkable features is its capacity to perform well on many natural language tasks in few-shot and zero-shot settings, which is unprecedented in the field of NLP [3,4]. As stated in OpenAI’s official blog, its training method is similar to InstructGPT, a version of GPT-3 fine-tuned by instructions [6,7]. GPT-3 is a foundation model for NLP, with 175 billion parameters. Foundation models for NLP are trained using language modeling objectives, including predicting the next token and doing cloze [8-10]. Since these objectives do not require additional human labeling, all that is needed is attention [11], a large text corpus, and enough computational resources for training large language models.

### Retrospective Data Sets

Our test questions comprised 2 groups. The first group was designed to assess the model’s ability to provide useful and accurate drug information. We selected 293 open-ended questions from medication consultations recorded in February 2023 by the Central Medical Center in Taiwan. A total of 103 questions related to chronic prescriptions and medication reservations were excluded. From the remaining 190 questions, 40 questions were randomly selected from both the general public and health care professionals (for a total of 80 questions) on traditional Chinese medicine (80/190, 42%). The test questions primarily covered 4 categories: medication information, dosage adjustment, drug-drug interactions, and intravenous injection compatibility (Multimedia Appendix 1, Table S1). The second part of the study further explored ChatGPT’s capability to handle specific types of questions; thus, we used 8 questions related to drug interactions between traditional Chinese and Western medicines. We specifically inquired about the interactions between aspirin and 8 types of Chinese medicines, including ginseng, safflower, licorice, astragalus, coptis, hawthorn, angelica, and ginkgo (Multimedia Appendix 1, Table S2).

### Prospective Testing

An assistant submitted both sets of questions (open-ended medication consultation questions and drug-herb interaction questions) to the ChatGPT interface, recording the initial response generated for each query.

In the first part of the study, an example of a question (translated from Chinese) is “What is the recommended meropenem dosage for patients with renal dysfunction?” An example of a ChatGPT response (translated from traditional Chinese) is “Meropenem dosing may require adjustment in patients with renal dysfunction, as the drug is primarily eliminated through the kidneys. Generally, for adult patients with renal impairment (creatinine clearance <50 mL/min), the recommended dose ranges between 500 mg and 1000 mg every 12 hours, depending on the severity of renal dysfunction” (Multimedia Appendix 1, Table S1). The second part of the study specifically focused on aspirin and its interactions with various Chinese medicines (eg, ginseng, safflower, licorice, astragalus, coptis, hawthorn, angelica, and ginkgo). Each question was structured similarly (Multimedia Appendix 1, Table S2).

For the responses generated by ChatGPT, we adopted the traditional double-review mechanism, enlisting 2 pharmacists, each with 10 years of professional experience, to independently conduct reviews. If a response was considered inappropriate, the reason for its inappropriateness was recorded. If discrepancies emerged between their evaluations, a third pharmacist was brought in for consultation. All disagreements were resolved through consensus to provide the final labeling. All the questions and answers generated, as well as the supplementary tables, were in Chinese. Ultimately, we used the built-in translation feature of ChatGPT-4 to directly translate the Chinese content into English.

### Statistical Analysis

We used descriptive statistics to illustrate the performance of ChatGPT, including the rate of inappropriate final labeling and the analysis of the reasons behind it. Categorical variables were analyzed using the chi-square test with SAS (version 9.4; SAS Institute). The level of statistical significance for all tests was set at a 2-sided *P* value of .05.

### Ethical Considerations

This survey study was deemed exempt from review by the Ethics Review Board of China Medical University because no personal identities were used.

## Results

### Real-World Medication Consultation Questions From the Medical Center

From the 190 real-world medication questions, 80 open-ended questions were randomly selected and evaluated by 2 pharmacists. The responses generated by ChatGPT were initially reviewed for appropriateness by the 2 pharmacists, with a discordance rate of 12.5% (10/80). The questions with inconsistent annotations (Multimedia Appendix 1, Table S1) underwent consensus resolution to obtain the final annotation of appropriateness or inappropriateness. We found that the appropriateness rate of ChatGPT’s responses to public drug consultation questions was higher compared to questions asked by health care providers in the hospital setting (31/51, 61% vs 20/51, 39%; *P*=.01; Table 1). Upon further analysis, ChatGPT gave incorrect answers in 12 of 29 cases (41%). In 5 of 29 instances (17%), ChatGPT’s responses lacked sufficient detail, and in another 12 of 29 cases (41%), it remained neutral without providing useful suggestions or information (Table 2).

**Table 1:** Pharmacist evaluation of ChatGPT’s appropriateness in responding to real-world medication consultation questions. Of the 80 responses, 51 were appropriate (64%) and 29 were inappropriate (36%; *P*=.01).

Source of the question	Appropriate responses (n=51), n (%)	Inappropriate responses (n=29), n (%)
Health care professionals	20 (39)	20 (69)
Patients	31 (61)	9 (31)

**Table 2:** Analysis of reasons for inappropriate responses to real-world medication consultation questions and drug-herb interaction questions.

ChatGPT responses	Real-world pharmaceutical consultation questions (n=29), n (%)	Drug-herb interaction questions (n=4), n (%)
Incorrect	12 (41)	1 (25)
Not detailed enough	5 (17)	2 (50)
Neutral and not useful	12 (41)	1 (25)

### Drug-Drug Interactions Between Chinese and Western Medicines

The final section of the test data set consisted of 8 questions selected by a pharmacist. Using aspirin as an example, we inquired about its potential interactions with ginseng, safflower, licorice, astragalus, coptis, hawthorn, angelica, and ginkgo. The results indicated that the rate of inconsistencies in the pharmacist’s annotations was 37.5%, (3/8) whereas ChatGPT displayed an inappropriateness rate of 50% (4/8) in its responses. Further analysis of ChatGPT’s answers revealed that the responses to the questions about whether aspirin could be used in combination with safflower, astragalus, coptis, and hawthorn lacked sufficient detail (Table 2).

## Discussion

### Appropriate Responses by ChatGPT

Previous research primarily used multiple-choice data sets to test language models, with a smaller number using real-world consultation questions [2,12]. Despite their proficiency in mimicking human language, large language models show limitations when answering open-ended questions, with the potential to generate biased, offensive, or incorrect responses [13]. A predominant challenge posed by the use of ChatGPT is the generation of “hallucinations,” as indicated in prior research [14]. The occurrence of hallucinatory outputs is not restricted to ChatGPT alone but constitutes a ubiquitous concern for all natural language generation (NLG) models. Research by Ji et al [15] has demonstrated that multiple factors contribute to the inception of these hallucinations within NLG models, but a range of methods for their mitigation have also been proposed. To effectively integrate this technology into practical use, we consider that both a dependable knowledge base and human oversight are indispensable. Our study found a lower accuracy rate for ChatGPT when addressing questions posed by health care professionals compared to those from the general public. We hypothesize that this could be due to the often specific and prognosis-related nature of health care professional inquiries, which may require access to textbooks or paid literature for comprehensive responses. Furthermore, these evidence-based medicine data sets, which serve as the foundation for such responses, often require subscriptions or are not freely available via open internet resources. Some clinical inquiries may not be covered within these databases, thus necessitating reliance on health care professionals’ clinical experience for complete answers. In contrast, the general public’s queries were largely generic and related to drug information or interactions, which can be easily accessed via free drug package inserts. For instance, in response to the consultation question “What precautions should be taken with Fosamax PLUS?” ChatGPT provided advice about consuming it on an empty stomach, remaining upright after consumption, and ensuring ample hydration. This response was deemed appropriate. However, ChatGPT did provide an incorrect response concerning a serious contraindication: it stated there was no intravenous interaction between ceftriaxone and calcium gluconate [16]. We surmise that this error resulted from the absence of compatibility data in the model’s training data set.

### Analysis of the Reasons for ChatGPT’s Incorrect Responses to Questions Regarding Drug-Herb Interactions

Our findings suggest that ChatGPT tends to produce analogous answers to similar queries. In our assessment of ChatGPT’s appropriate responses, most of them involved traditional Chinese medicinal materials that the general public is more familiar with, such as ginseng, licorice, angelica, and ginkgo. However, responses that were evaluated as inappropriate, such as those related to safflower, astragalus, coptis, and hawthorn, were deemed insufficient due to a lack of information, making it difficult to provide patients with clear recommendations. Therefore, we speculate that the machine learning model’s database may still lack sufficient information on traditional Chinese medicine.

Regarding the ChatGPT response to the safety and efficacy of using saffron with aspirin, there may not be enough research evidence. However, clinical evidence shows that the components of saffron can affect platelet function and inhibit blood clotting. Therefore, patients who are scheduled for surgery in the near future should avoid using saffron. Furthermore, combination prescriptions with saffron are very rare in clinical practice, and in some individuals who are sensitive to it, bleeding may occur. Additionally, saffron is expensive. The interaction rate of traditional Chinese medicine may indeed be affected by factors such as the patient’s health condition, age, and other medications they are taking, which could impact its practicality in clinical practice. These are factors that ChatGPT cannot discern.

We deem ChatGPT’s response on the interaction between aspirin and astragalus to be overly neutral. In traditional Chinese medicine theory, astragalus is characterized as a qi-tonifying herb with warming properties, believed to enhance qi, raise yang, nourish defensive qi, and consolidate the exterior. A study from China’s Shanxi Hospital suggests that concurrent use of astragalus injection and aspirin could potentially augment blood flow and elevate bleeding risk.

We consider the interaction responses between aspirin and coptis or hawthorn as inaccurate, attributable to the insufficiency of detail in ChatGPT’s responses. In traditional Chinese medicine theory, coptis (*huanglian*) is classified as a bitter and cold herb with primary functions to clear heat, dry dampness, purge fire, and detoxify. Mechanistically, it does not interfere with anticoagulants. *Huanglian* contains berberine, known for its effect on relaxing vascular smooth muscle, but it does not directly influence the anticoagulant mechanism of aspirin. Hawthorn belongs to the category of resolving food stagnation, promoting digestion, regulating qi, and dispersing blood stasis. Hawthorn contains various organic acids, which can help to contract the uterus, strengthen the heart, counteract arrhythmia, increase coronary blood flow, dilate blood vessels, lower blood pressure, and reduce blood lipids. Hawthorn has the function of promoting blood circulation, removing blood stasis, and relieving pain. It is used to treat postpartum abdominal pain and lochia retention caused by blood stasis or dysmenorrhea due to blood stasis. Therefore, it is not recommended to use it together with aspirin.

### Potential of Using ChatGPT for Pharmacy Education and Medication Consultation

ChatGPT is gaining attention for its ability to provide detailed and clear answers in many knowledge domains. The GPT model uses a text completion format to generate diverse responses by selecting the word with the highest probability. This demonstrates that knowledge-based jobs, previously believed to be immune to replacement by artificial intelligence, may now be within its capabilities [17,18]. Based on this research example, we hypothesize that such probability distribution may be used to assist pharmacy education and serve as a tool for public consultation. In terms of assisting pharmacy education, the responses given are based on the maximum probability distribution of the input text, which may represent the information that pharmacy students are most likely to encounter when searching for literature. We are optimistic that the answers generated by ChatGPT can be validated by pharmaceutical experts or clinical pharmacology teachers to identify blind spots in educational questions, provide appropriate feedback [19], and find ways to enhance the assessment of skills and behaviors so that we can develop in sync with potential changes in medical education and practice [20]. However, we are also concerned that not all pharmacists may have the ability or time to identify errors in the information provided by the chatbot [21].

In the 2 test sets, we found that the rate of inconsistency among pharmacists’ evaluations appeared to be higher in questions related to interactions between traditional Chinese and Western medicines (10/80, 12.5% vs 3/8, 37.5%; *P*=.06). In Taiwan, a higher proportion of pharmacists practice in the Western medicine domain compared to the field of traditional Chinese medicine. We reasonably infer that pharmacists may be more susceptible to the influence of information generated by ChatGPT in this subspecialty. We should be aware of the potential risks and harms that may arise from relying too heavily on ChatGPT for medical information and providing inaccurate information to health care providers [22].

As ChatGPT acquires its medical knowledge from online resources, we may expect substantial improvements in AI model performance with the development of technology and growing availability of open-access academic research. However, medical errors are not tolerated [23]. With this premise in mind, ChatGPT could be used to alleviate the burden of pharmacist consultation services for basic medication questions from the general public, providing faster and more immediate feedback that is not restricted by time or space. If used appropriately, we believe that ChatGPT can have a positive impact in these areas.

### Limitations

This study has several limitations. First, although ChatGPT is multilingual, we speculate that its responses in English may be more accurate due to a larger data pool. Second, limited by our research period, we used GPT-3.5 as the test model. The technical report released by OpenAI for GPT-4 highlights the substantial research efforts aimed at reducing hallucinations. It shows that GPT-4 produces fewer instances of hallucinatory output compared to earlier models. However, OpenAI acknowledges that the issue of hallucinations remains a current limitation of GPT-4 [24]. Therefore, our research findings retain their significance in addressing this concern. Third, while our questions were independent, some required background information, which might have induced baseline bias. Fourth, we emulated a busy drug consultation environment where incomplete background data might lead to less accurate ChatGPT responses. Assessing the potential biases and risks associated with these responses and developing additional methods or modules to mitigate and address any errors that may occur will be considered as our future research objectives.

### Conclusions

To our knowledge, studies discussing and analyzing the reasons for errors in ChatGPT’s responses are relatively scarce. We found that ChatGPT provided largely appropriate responses to simple medication questions as evaluated by pharmacists. However, for more complex questions related to individual patient scenarios, the answers may be inaccurate or vague, thereby making it challenging for the person asking the question to obtain the necessary information. As pharmacists, we recognize that many patients and health care professionals continue to depend on us for medication information and education. While we are optimistic about ChatGPT’s potential in assisting pharmacists in providing medication consultations to the public and aiding pharmacy educators in identifying gaps in student knowledge, our study suggests that we must remain cognizant of the risks associated with the provision of incorrect information.

## Appendix

Multimedia Appendix 1 Tables S1: ChatGPT responses to real-world pharmaceutical consultation questions and 3 pharmacists’ annotations; Table S2: ChatGPT responses to drug-herb interaction questions and 3 pharmacists’ annotations.

## Abbreviations

GPT: generative pretrained transformer
NLG: natural language generation
NLP: natural language processing
STROBE: Strengthening the Reporting of Observational Studies in Epidemiology
USMLE: United States Medical Licensing Examination
