# Supplementary material for: Examining Real-World Medication Consultations and Drug-Herb Interactions: ChatGPT Performance Evaluation
Source: JMIR Med Educ. 2023 Aug 21;9:e48433. doi: 10.2196/48433 (PMC10477918; doi:10.2196/48433)
Supplement: Multimedia Appendix 1 [file mededu_v9i1e48433_app1.docx]

Table of contents

[eTable 1. ChatGPT responses to real-world pharmaceutical consultation questions and three pharmacists' annotations 2](#_Toc131169717)

[eTable 2. ChatGPT responses for Drug-Herb Interactions questions and three pharmacists' annotations 71](#_Toc131169718)

**eTable 1. ChatGPT responses to real-world pharmaceutical consultation questions and three pharmacists' annotations**

| **No.** | **Classifications** | **Object** | **Question ^a^** | **Answer by ChatGPT ^a^** | **Assessments by pharmacist A/B** | **Confidence** ^b^ | **Assesment by pharmacist C** | **The cause of Inappropriate** |
| --- | --- | --- | --- | --- | --- | --- | --- | --- |
| 1 | DDI | Healthcare professionals | Can calcium channel blockers and oral calcium supplements be taken together? | Calcium channel blockers and oral calcium supplements can be taken together, but it is recommended to pay attention to an appropriate time interval to avoid interference with each other. Calcium channel blockers lower blood pressure and are usually used to treat hypertension and cardiovascular diseases. Oral calcium supplements, on the other hand, are used to prevent or treat calcium deficiency or conditions such as osteoporosis. If both of these medications are needed, it is best to leave a certain time interval between their intake, usually recommended to be more than 2 hours apart. | Appropriate/Appropriate | High | Appropriate | -- |
| 2 | DDI | Healthcare professionals | Do artificial sweeteners affect insulin? | Artificial sweeteners are low-calorie sweeteners used as a substitute for sugar in food. They are often considered a weight loss tool because they have fewer calories than sugar. However, some studies have shown that the use of artificial sweeteners may affect insulin secretion and metabolism. Insulin is a hormone secreted by the pancreas that regulates blood sugar levels. When we consume high-sugar foods, insulin promotes the entry of sugar into cells, which ultimately lowers blood sugar levels. However, some artificial sweeteners may stimulate insulin secretion, just like consuming high-sugar foods. This could lead to overproduction of insulin, eventually causing insulin resistance, which is a risk factor for type 2 diabetes. Therefore, if you have diabetes or other blood sugar control issues, it is best to consult a doctor before using artificial sweeteners. Additionally, artificial sweeteners should be combined with a healthy diet and exercise plan to maintain overall health. | Appropriate/Appropriate | High | Appropriate | -- |
| 3 | DDI | Healthcare professionals | What are the drug interactions between Atropine, Fentanyl, Furosemide, LORazepam, Midazolam, and Propofol? | Atropine and fentanyl may cause an increased risk of respiratory depression when used together. Furosemide may increase the risk of hypotension and dehydration when used with lorazepam or midazolam. It is important to monitor patients closely for these potential interactions and adjust dosages as needed. | Appropriate/Appropriate | High | Appropriate | -- |
| 4 | Dose adjustments | Healthcare professionals | What is the dosage about Meropenem in renal dysfunction patients? | The dosing of Meropenem may need to be adjusted in patients with renal dysfunction, as the drug is primarily eliminated by the kidneys. In general, the recommended dose in adult patients with renal impairment (creatinine clearance <50 mL/min) is 500 mg to 1000 mg every 12 hours, depending on the severity of renal dysfunction. The dose may be adjusted further based on the patient's clinical response and the results of therapeutic drug monitoring. | Inappropriate/Inappropriate | High | Inappropriate | Chatgpt gave an incorrect answer |
| 5 | Dose adjustments | Healthcare professionals | What is the dosage about tramadol in liver dysfunction patients? | In general, the recommended starting dose of tramadol in patients with mild to moderate liver dysfunction (Child-Pugh Class A or B) is 50 mg every 12 hours, and the maximum daily dose should not exceed 200 mg. Tramadol is not recommended for use in patients with severe liver dysfunction (Child-Pugh Class C) due to the risk of drug accumulation and toxicity. | Appropriate/Appropriate | High | Appropriate | -- |
| 6 | Dose adjustments | Healthcare professionals | What is the maximum dose of dicloxacillin? | The maximum recommended dose of dicloxacillin depends on the patient's age, weight, and the condition being treated. In adults, the usual maximum daily dose of dicloxacillin is 4 grams per day, divided into 2 to 4 equal doses. However, the maximum dose can vary depending on the severity of the infection and other individual factors. It is important to follow the dosage and administration instructions provided by the healthcare provider or as indicated on the medication label. | Inappropriate/Inappropriate | High | Inappropriate | Chatgpt gave an incorrect answer |
| 7 | Dose adjustments | Healthcare professionals | What is the dosage about Sulfamethoxazole/Trimethoprim in renal dysfunction patients? | The usual adult dose of sulfamethoxazole/trimethoprim (a combination medication) is 800mg sulfamethoxazole per day for urinary tract infections. However, if the patient's CrCl is less than 30 mL/min, the dose should be reduced to 400mg sulfamethoxazole per day or less. | Inappropriate/Appropriate | Low | Inappropriate | Chatgpt response is not detailed enough |
| 8 | Dose adjustments | Healthcare professionals | What is the dosage of IV ampiciiln in children? | The dosage of ampicillin in children depends on the child's age, weight, and the condition being treated. The usual intravenous (IV) dose is 100-200 mg/kg per day, given in divided doses every 6-8 hours. | Inappropriate/Inappropriate | High | Inappropriate | Chatgpt gave an incorrect answer |
| 9 | Dose adjustments | Healthcare professionals | Patient's Glomelular filter rate 36ml/min, CRE1.45mg/dL, how to adjust the dosage for pylori infection three-in-one ulcer medication? | In general, for patients with renal impairment (glomerular filtration rate < 60 ml/min), the medication dosage should be adjusted based on their kidney function. In this case, the patient's GFR is 36 ml/min, which suggests moderate to severe renal impairment. Therefore, the medication dosage should be adjusted as follows: The dosing frequency may need to be decreased, or the medication may be given every other day instead of daily. The dosage amount may need to be decreased to avoid toxicity and accumulation of the medication in the patient's system. The duration of treatment may need to be extended to compensate for the reduced dosage. It's important to note that the dosage adjustment should be made based on the patient's individual renal function and the medication's pharmacokinetics. Therefore, consulting a healthcare professional is necessary before making any adjustments. | Appropriate/Appropriate | High | Appropriate | -- |
| 10 | Dose adjustments | Healthcare professionals | What is the dosage of Sevatrim injection( Sulfamethoxazole(SMX) 400 mg and Trimethoprim(TMP) 80 mg) for renal insufficiency patients? | The dosage of Sevatrim injection (Sulfamethoxazole 400 mg and Trimethoprim 80 mg) for patients with renal insufficiency depends on the severity of the condition and the patient's creatinine clearance. The recommended dosage for patients with creatinine clearance less than 15 mL/min is 50% of the normal dose given every 24 hours. However, it is important to consult a healthcare provider before taking any medication. | Inappropriate/Inappropriate | High | Inappropriate | Chatgpt response is not detailed enough |
| 11 | Dose adjustments | Healthcare professionals | How should teicoplanin be administered in patients with renal impairment (CLCr <40)? | For patients with impaired renal function, the dosage and frequency of teicoplanin administration need to be adjusted. It is recommended to consult a doctor immediately to determine the correct dosage and frequency of use. Generally, for patients with impaired renal function, the dose of teicoplanin needs to be reduced and the dosing interval prolonged. Depending on the specific condition of the patient, the doctor may need to further adjust the dosage and administration method. | Inappropriate/Inappropriate | High | Inappropriate | ChatGPT remained neutral and did not provide any suggestions |
| 12 | Dose adjustments | Healthcare professionals | What is the maximum daily dose of fluconazole 50mg/cap oral antimicrobial drugs in our hospital? | Fluconazole is an antifungal drug commonly used to treat fungal infections such as candidiasis. The dosage will vary depending on the condition and the patient's situation, and it is recommended to use under the guidance of a doctor. In general, the recommended daily oral dose for adults does not exceed 400 milligrams, taken once or twice daily. It is advised to follow the doctor's prescription and the instructions on the medication label. Additionally, if there are any symptoms or discomfort, it is important to inform the doctor promptly and adjust the medication dosage as directed. | Appropriate/Appropriate | High | Appropriate | -- |
| 13 | medication information | Healthcare professionals | Is there any precaution for patient with G6PD to use tamsulosin(oral dissolve tablet)? | It is important for patients with G6PD deficiency to consult with their healthcare provider before taking any new medication, including tamsulosin, to ensure it is safe for them to use. Tamsulosin is a medication used to treat urinary symptoms caused by an enlarged prostate, and it is important to note that it may interact with other medications and have potential side effects. Therefore, it is important to follow the advice of a healthcare professional before using tamsulosin or any other medication. | Inappropriate/Inappropriate | High | Inappropriate | ChatGPT remained neutral and did not provide any suggestions |
| 14 | medication information | Healthcare professionals | How to prescribe Lenograstim 100mcg/Vial for Malignant neoplasm of anterior mediastinum? | Here is a possible prescription for Lenograstim 100mcg/vial for malignant neoplasm of anterior mediastinum: Dosage: 263 mcg/day subcutaneously (SC) or 263 mcg/day intravenously (IV) for 14 consecutive days. Instructions: Reconstitute the Lenograstim 100mcg/vial with 1 mL of sterile water for injection. Withdraw 2.63 mL (263 mcg) of the reconstituted solution using a sterile syringe. Administer the solution subcutaneously or intravenously as directed. Repeat the administration daily for 14 consecutive days. Monitor the patient for any adverse reactions or signs of infection during the treatment. Note: The dose and duration of treatment may vary depending on the patient's age, weight, medical history, and other factors. Always follow the prescribing physician's instructions and consult a healthcare professional if you have any questions or concerns. | Inappropriate/Inappropriate | High | Inappropriate | Chatgpt gave an incorrect answer |
| 15 | medication information | Healthcare professionals | Patient response to Celebrex(Celecoxib 200 mg/Cap) with cold sweating, what is the best medical prescription? | The healthcare professional may recommend an alternative medication or adjust the dosage accordingly. | Appropriate/Appropriate | High | Appropriate | -- |
| 16 | medication information | Healthcare professionals | What is the initial medication for hyperhidrosis? | The first choice anticholinergic oral medication for hyperhidrosis is usually glycopyrrolate (Robinul). | Appropriate/Appropriate | High | Appropriate | -- |
| 17 | medication information | Healthcare professionals | How to dilute Sevatrim( Sulfamethoxazole/Trimethoprim) 480 mg/5 mL/Amp for injection? | If a healthcare provider has prescribed Sevatrim for intravenous infusion, the following steps may be taken to dilute the medication: Calculate the appropriate dose based on the patient's weight and the indication for treatment. Obtain a sufficient volume of sterile diluent, such as 0.9% Sodium Chloride Injection or 5% Dextrose Injection, to achieve the desired concentration. Withdraw the required volume of Sevatrim injection from the vial and add it to the diluent. Mix the solution gently to ensure proper dilution. Administer the solution as directed by the healthcare provider, usually over a period of 60-90 minutes. | Inappropriate/Appropriate | Low | Inappropriate | Chatgpt response is not detailed enough |
| 18 | medication information | Healthcare professionals | What are the safe precaution for the patient that had allergy history of hyperthyroidism in amiodarone use? | Generally, some of the safe precautions that may be recommended by a healthcare professional are: The patient should be closely monitored for any signs of an allergic reaction such as rash, hives, difficulty breathing, or swelling. The patient should inform their healthcare provider about their allergy history of amiodarone and hyperthyroidism. The patient should take the medication exactly as prescribed by their healthcare provider and not alter the dosage or stop taking the medication without consulting their doctor. Regular thyroid function tests should be performed to monitor the patient's thyroid levels, especially if they have a history of hyperthyroidism. The patient should avoid taking other medications that may interact with amiodarone, and they should inform their healthcare provider about any new medication they plan to take. The patient should avoid consuming grapefruit or grapefruit juice as it may increase the risk of side effects. The patient should avoid exposure to excessive sunlight or use protective clothing or sunscreen as amiodarone may increase sensitivity to sunlight. | Appropriate/Appropriate | High | Appropriate | -- |
| 19 | medication information | Healthcare professionals | Is Ketorolac injection suitable for a 1-year-old child with fever? | Ketorolac is a nonsteroidal anti-inflammatory drug (NSAID) used to relieve moderate to severe pain, but it is generally not recommended for fever. The use of Ketorolac in children is limited because it may have potential adverse effects on their kidney function and gastrointestinal tract. According to the Ketorolac prescribing information, it should not be used in children under 12 years of age unless directed otherwise. Therefore, Ketorolac injection is not suitable for use in a 1-year-old child. If your child has a fever, it is best to consult a pediatrician for advice to obtain appropriate recommendations and treatment. | Appropriate/Appropriate | High | Appropriate | -- |
| 20 | medication information | Healthcare professionals | What are the side effects of using Berotec (fenoterol 100mcg) more than 8 times a day? | Exceeding the recommended usage of Berotec (fenoterol 100mcg) can increase the risk of side effects. Some of the potential side effects include tremors, headaches, dizziness, palpitations, increased heart rate, and decreased potassium levels. In rare cases, it can also cause more serious side effects such as chest pain, irregular heartbeat, and allergic reactions. It is important to follow the recommended dosage and consult a healthcare professional if you experience any concerning symptoms. | Appropriate/Appropriate | High | Appropriate | -- |
| 21 | medication information | Healthcare professionals | Can patients with sulfonamide allergy use Zonisamide? | Zonisamide is a sulfonamide derivative and may cause cross-sensitivity reactions in patients with sulfonamide allergy. Therefore, patients with a history of sulfonamide allergy should use zonisamide with caution and only under the supervision of a healthcare professional who can monitor for any potential allergic reactions. | Appropriate/Appropriate | High | Appropriate | -- |
| 22 | medication information | Healthcare professionals | What is the pharmacological mechanism of Cymbalta(Duloxetine) in the treatment of fibromyalgia? | Duloxetine is a medication that primarily works by regulating the balance of neurotransmitters to improve mood and alleviate pain. For fibromyalgia, Duloxetine works mainly through the following pharmacological actions: Inhibiting the reuptake of norepinephrine and serotonin to increase their concentration in the central nervous system, thereby increasing the pain threshold and reducing pain sensations. Regulating the mood by inhibiting the reuptake of serotonin and norepinephrine, which helps to alleviate emotional symptoms such as depression and anxiety and improve the overall psychological state of the patient. Inhibiting pain transmission in the spinal cord by reducing the discharge of nociceptive neurons, which reduces pain sensations. Overall, Duloxetine can simultaneously alleviate pain and emotional symptoms, thereby improving the quality of life and work of patients with fibromyalgia. | Appropriate/Appropriate | High | Appropriate | -- |
| 23 | medication information | Healthcare professionals | Can Posaconazole be used in a 2-year-old child? | The age limit for the use of Posaconazole varies depending on the intended use. If you want to know whether Posaconazole is safe and effective for treating specific diseases in children aged 2 years, consult a pediatrician. They can assess the risks and benefits of using Posaconazole based on the child's condition and physical health, and provide appropriate recommendations. | Inappropriate/Inappropriate | High | Inappropriate | ChatGPT remained neutral and did not provide any suggestions |
| 24 | medication information | Healthcare professionals | How to store EFLOR powder? | EFLOR powder should be stored at room temperature, avoiding moisture and away from direct sunlight. Do not store the medication in a refrigerator or freezer. Please store and use the medication according to the instructions on the package insert. If you have any questions, please consult your doctor or pharmacist. | Inappropriate/Appropriate | Low | Appropriate | -- |
| 25 | medication information | Healthcare professionals | Treatment regimen for penicillin in syphilis | Treatment of syphilis typically involves the use of penicillin antibiotics, with the standard treatment regimen consisting of weekly penicillin injections for 3 weeks, or for 2 weeks with weekly injections. The specific treatment plan depends on the severity of the condition and the patient's circumstances. It is recommended to consult a doctor for a personalized treatment plan before undergoing treatment. | Inappropriate/Inappropriate | High | Inappropriate | ChatGPT remained neutral and did not provide any suggestions |
| 26 | medication information | Healthcare professionals | How to administer Nexium through a feeding tube? | Nexium is an oral medication typically administered to patients in tablet form. If it is necessary to administer Nexium via a feeding tube, it is recommended to crush the tablet into a fine powder and dissolve it in water, stirring well before administering it via the feeding tube. The specific method of administration should be guided by healthcare professionals, as improper administration may cause discomfort or harm to the patient. Before administering via a feeding tube, it is recommended to thoroughly read the Nexium user manual or consult a physician or pharmacist for guidance to ensure the safe and effective use of the medication. | Inappropriate/Inappropriate | High | Inappropriate | Chatgpt gave an incorrect answer |
| 27 | medication information | Healthcare professionals | What are the medications that inhibit lactation? | The commonly used medications for inhibiting lactation on the market are "bromocriptine" and "cabergoline". These drugs are often used to treat abnormal lactation caused by high levels of prolactin or to suppress lactation after breastfeeding has stopped. These medications are prescription drugs and should be used under the guidance of a doctor. Please follow the doctor's instructions when using them. | Appropriate/Appropriate | High | Appropriate | -- |
| 28 | medication information | Healthcare professionals | What is the meaning of pregnancy category C for Esomeprazole? | The drug pregnancy categorization system is used to describe the level of risk to the fetus when using a certain medication during pregnancy. According to the US FDA categorization system, drugs are classified into five categories: A, B, C, D, and X, where category C indicates that the drug has shown adverse effects on the fetus in animal studies, but there is not enough evidence from human clinical trials to prove its risk to the fetus. For pregnant women, doctors usually weigh the benefits and risks and make a judgment on whether to use category C drugs based on the specific circumstances of the patient, or consider other safe alternative medications. Esomeprazole is a category C drug that should be used with caution during pregnancy, and it is recommended to use it under the guidance of a doctor. | Appropriate/Appropriate | High | Appropriate | -- |
| 29 | medication information | Healthcare professionals | Can Fosrenol chewable tablet grind into powder and taken together with food? | Fosrenol chewable tablet is a type of phosphate binder used to lower blood phosphate levels. According to the official drug information, Fosrenol should be chewed and then rinsed with water to ensure proper absorption and reduce oral and gastrointestinal discomfort. Therefore, if you want to grind Fosrenol into powder and take it with food, it is recommended to do so under the guidance of a doctor or pharmacist to ensure the effectiveness and safety of the medication. | Appropriate/Appropriate | High | Appropriate | -- |
| 30 | Parenteral compatibility | Healthcare professionals | How is the Y-site compatibility of Amiodarone and Calcium Gluconate? | Amiodarone and Calcium Gluconate are compatible when given through the same intravenous line (Y-site compatibility). However, compatibility of the two drugs may vary based on different factors such as patient's conditions, drug formulation, and concentration of the drugs. It is always recommended to consult a pharmacist or the drug compatibility reference before administering multiple drugs together through the same intravenous line. | Appropriate/Appropriate | High | Appropriate | -- |
| 31 | Parenteral compatibility | Healthcare professionals | How is the Y-site compatibility of amiodarone and heparin sodium? | Amiodarone and Heparin sodium are generally compatible when given through the same intravenous line (Y-site compatibility). However, compatibility of the two drugs may vary based on different factors such as patient's conditions, drug formulation, and concentration of the drugs. It is always recommended to consult a pharmacist or the drug compatibility reference before administering multiple drugs together through the same intravenous line. | Inappropriate/Inappropriate | High | Inappropriate | Chatgpt gave an incorrect answer |
| 32 | Parenteral compatibility | Healthcare professionals | How is the Y-site compatibility of ceftriaxone and Calcium Gluconate? | Ceftriaxone and Calcium Gluconate are generally considered compatible when given through the same intravenous line (Y-site compatibility). However, compatibility may vary based on different factors such as patient's conditions, drug formulation, and concentration of the drugs. It is recommended to consult a pharmacist or a drug compatibility reference before administering multiple drugs together through the same intravenous line. | Inappropriate/Inappropriate | High | Inappropriate | Chatgpt gave an incorrect answer |
| 33 | Parenteral compatibility | Healthcare professionals | What kind diluted solutions is available for Meropenem powder injection? | Meropenem powder for injection is typically reconstituted with sterile water for injection to produce a solution for intravenous administration. The reconstituted solution is then further diluted with a compatible infusion solution, such as 0.9% sodium chloride injection, 5% dextrose injection, or lactated Ringer's injection, to a final concentration ranging from 0.25 mg/mL to 2 mg/mL, depending on the dose and the patient's condition. | Inappropriate/Inappropriate | High | Inappropriate | Chatgpt gave an incorrect answer |
| 34 | Parenteral compatibility | Healthcare professionals | Is diluted solution such as D5W in 0.33% NaCl compatible with Lorazepam injection? | Lorazepam injection is compatible with a variety of diluents, including normal saline, dextrose 5% in water (D5W), and Ringer's lactate. However, the specific diluent to be used may depend on the specific manufacturer's recommendations and the patient's clinical condition. It is important to follow the manufacturer's instructions and the healthcare provider's orders when preparing and administering Lorazepam injection. | Inappropriate/Appropriate | Low | Inappropriate | Chatgpt response is not detailed enough |
| 35 | Parenteral compatibility | Healthcare professionals | What kind of dilute solution for diazepam injection? | Diazepam Injection is available in a concentrated form of 5 mg/mL. It should be diluted with an appropriate IV fluid before administration. The usual dilution range for Diazepam Injection is 1:1 to 1:5. The recommended diluent for Diazepam Injection is 0.9% Sodium Chloride Injection or 5% Dextrose Injection. Other IV fluids, such as Ringer's Lactate or Dextran solutions, are not recommended for dilution. It is important to follow the proper dilution instructions provided by the manufacturer or healthcare provider to ensure the safe and effective use of Diazepam Injection. | Inappropriate/Appropriate | Low | Appropriate | Pharmacist A gave an incorrect answer |
| 36 | Parenteral compatibility | Healthcare professionals | Vancomycin 500mg IV Q8hI trough:19.9，how should I adjust for the suitable trough concentration? | According to this trough concentration value, the dose of Vancomycin may need to be adjusted. The specific adjustment depends on individual patient factors, such as renal function and age. It is recommended to consult with a doctor immediately to determine the correct dose adjustment. | Inappropriate/Inappropriate | High | Inappropriate | ChatGPT remained neutral and did not provide any suggestions |
| 37 | Parenteral compatibility | Healthcare professionals | Vancomycin 1000mg Q12H*4 doses, trough level: 13.9mg/L, distribution 8 hrs. Renal function is getting worse | According to this trough concentration value, the dose of Vancomycin may need to be adjusted. The specific adjustment depends on individual patient factors, such as renal function and age. It is recommended to consult with a doctor immediately to determine the correct dose adjustment. | Inappropriate/Inappropriate | High | Inappropriate | ChatGPT remained neutral and did not provide any suggestions |
| 38 | Parenteral compatibility | Healthcare professionals | Is pralidoxime compatible with diluents for organophosphate antidotes? | The preparation and use of Pralidoxime diluent may vary depending on the recommendations of different healthcare institutions and manufacturers, therefore it is recommended to read the drug's instructions or consult with healthcare professionals for related preparation and usage guidelines before use. When diluting and using Pralidoxime, it is important to pay attention to its compatibility with other medications to avoid adverse reactions and events. | Inappropriate/Inappropriate | High | Inappropriate | ChatGPT remained neutral and did not provide any suggestions |
| 39 | Parenteral compatibility | Healthcare professionals | Can bfluid and 10% dextrose use together? | bfluid and 10% glucose injection can be used together. bfluid is a fluid replacement supplement used to replenish fluids and electrolytes, while 10% glucose injection is a nutritional injection. In some cases, healthcare professionals may recommend using both drugs together to achieve treatment goals. However, to confirm the safety of using bfluid and 10% glucose injection, it is recommended to consult with a doctor or pharmacist for advice. | Appropriate/Appropriate | High | Appropriate | -- |
| 40 | Parenteral compatibility | Healthcare professionals | Can 3-way tube use in TPN and medicine given together? | TPN is a therapeutic method of providing nutrition and medication through a central venous catheter. During TPN treatment, medication can be administered through a 3-way tubing, but the following procedures must be carefully followed:  Before administering any medication, it should be confirmed whether the medication can be mixed with the TPN solution.  Before starting to inject medication, the 3-way tubing must be flushed to prevent interactions between different medications.  Each time medication is added, attention must be paid to compatibility, concentration, and injection rate to avoid medication precipitation or adverse reactions.  Immediately after adding medication, the 3-way tube should be tightly closed to prevent air from entering the TPN solution, and should be discontinued immediately after injection.  During TPN treatment, seeking guidance and advice from professional healthcare personnel is recommended to ensure the safety and effectiveness of the treatment. | Appropriate/Inappropriate | Low | Inappropriate | ChatGPT remained neutral and did not provide any suggestions |
| 41 | DDI | Patients | My husband is having amlodipine and alprazolam. Can he take these medications together at the same time? | As an AI language model, I do not have the complete medical history of your husband, so I cannot provide a definitive answer to this question. However, it is always recommended to consult a qualified healthcare professional before combining medications, especially if there are potential interactions between them. Amlodipine is a calcium channel blocker used to treat high blood pressure, and alprazolam is a benzodiazepine used to treat anxiety disorders. They can interact and cause side effects such as dizziness, drowsiness, and difficulty concentrating. Therefore, it is important to seek medical advice to determine if it is safe for your husband to take these medications together. | Inappropriate/Inappropriate | High | Inappropriate | Chatgpt gave an incorrect answer |
| 42 | DDI | Patients | I had hemoptysis recently.I just taken one Avilox tablet and I am using erythromycin. May I ask what is the effect of Avelox, and is there a risk of coughing up blood when used together? | Avelox is a fluoroquinolone-type antibiotic, it is used to treat bacterial infection disease. It inhibits DNA synthesis and cell division which function as bactericidal. Avelox itself unable to cause blood coughing, but with erythromycin, it might increase the bleeding risk, including lung bleeding which, might cause coughing blood. Therefore when both these drugs are taken together, please inform the doctor. The doctor might suggest discontinuing one of the drug use or adjusting the dosage of the drug to decrease the bleeding risk. | Appropriate/Appropriate | High | Appropriate | -- |
| 43 | DDI | Patients | Can Cetirizine (liquid) Syrup 1mg/mL 60mL/Bot Peace (Triprolidine HCl 0.25 mg and Pseudoephedrine HCl 6 mg) syrup 60mL/Bot (compound) be taken at the same time? | It is not recommended to take both Cetirizine syrup and Peace syrup at the same time without consulting a doctor or a pharmacist. Both the syrups contain different active ingredients, and taking them together may lead to unwanted side effects or interactions. It is essential to follow the prescribed dosage and instructions provided by the healthcare provider for safe and effective treatment. | Appropriate/Appropriate | High | Appropriate | -- |
| 44 | DDI | Patients | What happens if aspirin is taken during a course of anticoagulant therapy? | Taking aspirin during a course of anticoagulant therapy can increase the risk of bleeding, which can be dangerous and potentially life-threatening. | Appropriate/Appropriate | High | Appropriate | -- |
| 45 | DDI | Patients | Is it safe to take urology and cardiovascular medications with B-complex vitamins, Q10, and fish oil supplements? | When taking urology and cardiovascular medication along with B-group vitamins, Q10, and fish oil supplements, it is advisable to exercise caution and consult a doctor or pharmacist for advice. This is because the composition of these drugs and supplements may affect each other, leading to adverse reactions or reduced effectiveness. For example, some cholesterol-lowering and blood pressure-lowering drugs may reduce the effectiveness of fish oil, while anticoagulant drugs and B-group supplements may increase the risk of bleeding. Additionally, Q10 may reduce the effectiveness of blood pressure-lowering drugs. Therefore, when using these medications and supplements simultaneously, it is recommended to consult a doctor or pharmacist, let them know about all the medications and supplements you are taking, and let them guide you on how best to combine these medications and supplements to ensure safety and effectiveness." | Inappropriate/Inappropriate | High | Inappropriate | ChatGPT remained neutral and did not provide any suggestions |
| 46 | DDI | Patients | What happens if alprazolam is taken during a course of paxlovid therapy? | co-administration of Paxlovid and alprazolam can lead to increased blood levels of alprazolam, which can increase the risk of adverse effects, such as excessive sedation, respiratory depression, or impaired motor coordination. Therefore, it is important to exercise caution when using these medications together and to closely monitor for any signs of adverse effects. | Inappropriate/Appropriate | Low | Inappropriate | Chatgpt response is not detailed enough |
| 47 | DDI | Patients | Is it suitable to take fish oil when I am currently taking aspirin and Plavix(Clopidogrel)? | It is generally recommended to consult with your doctor or a qualified healthcare professional before taking any supplements, including fish oil, especially if you are already taking other medications such as aspirin and Plavix (Clopidogrel).  Fish oil supplements contain omega-3 fatty acids, which may have a blood-thinning effect, similar to aspirin and Plavix. Taking fish oil in combination with these medications may increase your risk of bleeding.  Your doctor or healthcare provider will be able to advise you on whether taking fish oil is suitable for you, based on your individual health status, medical history, and any other medications you may be taking. They may also recommend adjusting the dosage of your current medications or suggest an alternative supplement that is safe for you to take. | Appropriate/Appropriate | High | Appropriate | -- |
| 48 | DDI | Patients | I am currently having Doxaben(doxazosin), can I use (Viagra) sildenafil during the treatment? | Doxazosin and sildenafil can interact with each other and may cause a significant drop in blood pressure, which can lead to serious side effects such as dizziness, fainting, or even a heart attack or stroke. Therefore, it is essential to inform your healthcare provider about all the medications you are taking, including over-the-counter drugs, herbal supplements, and vitamins, before taking sildenafil or any other medication.  Your healthcare provider can evaluate your overall health status, assess any potential risks, and determine whether sildenafil is appropriate for you while taking doxazosin. They may adjust the dosage or recommend alternative treatments to reduce the risk of interactions and ensure your safety. | Appropriate/Inappropriate | Low | Inappropriate | ChatGPT remained neutral and did not provide any suggestions |
| 49 | DDI | Patients | Can I take Domperidone(liquid) after Metoclopramide was administered in the emergency department? | It is recommended to wait at least 2 hours after taking Metoclopramide before taking Domperidone to avoid drug interactions. Metoclopramide and Domperidone are both medications that promote gastrointestinal motility, and using them at the same time may increase the risk of adverse reactions. If it is necessary to continue using these two medications, it is recommended to seek the advice of a doctor or pharmacist. | Inappropriate/Inappropriate | High | Inappropriate | ChatGPT gave an incorrect answer |
| 50 | DDI | Patients | Can Edoxaban 30mg/Tab be taken together fish oil at the same time? | Yes. However, fish oil should be noted that the risk of bleeding may increase as it has an anticoagulant effect. Therefore, it is recommended to consult with a doctor or pharmacist before use to confirm whether it is suitable to use these two medications simultaneously. | Appropriate/Appropriate | High | Appropriate | -- |
| 51 | medication information | Patients | Whether it is safe to breastfeed while using SCOPOLAMINE patches | It is always recommended to consult a healthcare professional for specific medical advice. The safety of using scopolamine patches while breastfeeding has not been well established and more information is needed. | Inappropriate/Inappropriate | High | Inappropriate | Chatgpt gave an incorrect answer |
| 52 | medication information | Patients | What is the side effect about Isosorbide-5-mononitrate 20mg/Tab? | Common side effects of isosorbide-5-mononitrate include headache, dizziness, flushing (feeling of warmth), and nausea. Less common side effects may include chest pain, fast or irregular heartbeat, and swelling of the ankles or legs. Some people may experience no side effects or only minor ones. Contact your doctor if you experience any severe or persistent side effects while taking isosorbide-5-mononitrate. | Appropriate/Appropriate | High | Appropriate | -- |
| 53 | medication information | Patients | Do I need to stop taking Rivaroxaban before tooth extraction? | It is recommended to consult with your healthcare provider or dentist before tooth extraction if you are taking Rivaroxaban. They will evaluate your individual risk factors and provide guidance on whether or not you should stop taking the medication before the procedure. | Appropriate/Inappropriate | Low | Inappropriate | ChatGPT remained neutral and did not provide any suggestions |
| 54 | medication information | Patients | What precautions about Fosamax PLUS? | Fosamax PLUS is a medication that contains both alendronate sodium and cholecalciferol (vitamin D3) and is used to treat osteoporosis. Here are some important precautions to keep in mind when taking Fosamax PLUS: Follow the dosing instructions carefully: Fosamax PLUS should be taken once a week, and it's important to follow the dosing instructions provided by your doctor. Do not take more than one tablet per week, and take the tablet with a full glass of water. Take Fosamax PLUS on an empty stomach: Fosamax PLUS should be taken at least 30 minutes before the first food, beverage, or medication of the day. This is important because food, beverages, and medications can interfere with the absorption of alendronate. Stay upright after taking Fosamax PLUS: To help prevent irritation to the esophagus, it's important to remain upright (sitting or standing) for at least 30 minutes after taking Fosamax PLUS. Drink plenty of water: To help prevent irritation to the esophagus, it's important to drink a full glass of water with Fosamax PLUS and to continue drinking plenty of fluids throughout the day. Let your doctor know if you have any medical conditions: Before taking Fosamax PLUS, let your doctor know if you have any medical conditions, such as kidney problems, vitamin D deficiency, or any digestive problems, as these may affect your ability to take this medication. Inform your doctor of other medications you are taking: Fosamax PLUS may interact with certain medications, such as calcium supplements, antacids, or other medications used to treat osteoporosis. Let your doctor know of all the medications and supplements you are taking. Report any side effects to your doctor: Fosamax PLUS can cause side effects, such as stomach upset, heartburn, or difficulty swallowing. If you experience any side effects, inform your doctor promptly. | Appropriate/Appropriate | High | Appropriate | -- |
| 55 | medication information | Patients | May I ask if Betmiga 50mg/tablet can be split in half? | According to the official drug information of Betmiga (a beta-3 adrenergic agonist), it is recommended to swallow the whole tablet and not to chew or split it in half | Appropriate/Appropriate | High | Appropriate | -- |
| 56 | medication information | Patients | How to use effervescent tablet? | The way to use foaming tablets is slightly different due to varies of brands and drug compounds. It is recommended to read the instructions on the product package or consult a physician or pharmacist before use. In general, the steps of using foam ingot are as follows: Prepare a cup of water or other specified solvents, and add to the cup according to the instructions on the instructions. Put the foaming tablet into the cup, give it a gentle stir, and wait for it to foam. When the foam is subsided, then stir again gently to ensure the tablet is completely dissolved. Drink directly or use according to the instructions. Precautions: Please read the instructions before use and follow the recommended dosage. Do not chew, split or crush the effervescent tablet. In case of allergic reaction, stop using it immediately and seek medical attention. If you have any questions, please consult your doctor or pharmacist. | Appropriate/Appropriate | High | Appropriate | -- |
| 57 | medication information | Patients | Magnesium Oxide 250mg/Tab adverse event? | Magnesium oxide is a mineral supplement that is commonly used to treat or prevent low levels of magnesium in the body. Adverse events associated with magnesium oxide are generally rare, but can occur in some individuals. Some of the common adverse events associated with magnesium oxide include: Diarrhea: Taking too much magnesium oxide can lead to loose stools and diarrhea. Upset stomach: Some people may experience stomach discomfort or nausea when taking magnesium oxide. Dehydration: Magnesium oxide can cause dehydration in some individuals, especially those with pre-existing kidney problems. Hypotension: In some rare cases, magnesium oxide can cause low blood pressure or hypotension. Cardiac arrest: Although extremely rare, excessive magnesium supplementation can lead to cardiac arrest. It is important to consult with your healthcare provider before starting any new supplement, including magnesium oxide, to determine the appropriate dosage and to discuss potential adverse events. Additionally, if you experience any severe or persistent symptoms while taking magnesium oxide, seek medical attention immediately. | Appropriate/Appropriate | High | Appropriate | -- |
| 58 | medication information | Patients | Why doctor prescribes CIPROfloxacin 500mg/Tab and Curam(Tablet) 1g/Tab together? | As an AI language model, I cannot provide specific medical advice. However, it is possible that a doctor may prescribe Ciprofloxacin and Curam together to treat a bacterial infection. Both medications belong to different classes of antibiotics and work differently to kill bacteria. Combining two antibiotics may provide a broader spectrum of coverage, increase effectiveness, and reduce the risk of bacterial resistance. It is essential to follow your doctor's instructions and complete the full course of treatment as prescribed. | Appropriate/Appropriate | High | Appropriate | -- |
| 59 | medication information | Patients | What are the common side effects of azithromycin(tablet) 250mg/tab? | The common side effects of azithromycin (tablet) 250mg/tab include nausea, vomiting, diarrhea, abdominal pain, headache, dizziness, and skin rash. Some people may also experience allergic reactions, such as difficulty breathing, swelling of the face, lips, tongue or throat, and hives. It is important to consult a doctor if any of these side effects persist or worsen. | Appropriate/Appropriate | High | Appropriate | -- |
| 60 | medication information | Patients | What is the best way to give Smecta to my children? | Smecta is a medication used to treat diarrhea and other gastrointestinal disorders. It contains the active ingredient diosmectite, which works by binding to toxins and bacteria in the gut, reducing inflammation and promoting the formation of a protective layer in the intestine. Smecta is usually taken orally, by mixing the contents of a sachet with water or another liquid. Here are the general steps to take Smecta: Open the sachet and pour the contents into a glass. Add a small amount of water or other liquid (about 50 mL) and stir until the powder is completely dissolved. Add more water or liquid to the glass to bring the total volume to 100 mL. Stir again and drink the entire mixture. It is usually recommended to take Smecta after meals, 2-3 times a day. It is important to follow the dosage and frequency instructions provided by your doctor or pharmacist. Smecta is generally well-tolerated, but like all medications, it may cause side effects such as constipation or nausea in some people. If you have any concerns or experience any adverse effects, contact your healthcare provider. | Appropriate/Appropriate | High | Appropriate | -- |
| 61 | medication information | Patients | I have difficulties chewing the Ferric chewable tab, how can I use this medication? | As an AI language model, I cannot provide medical advice or diagnose medical conditions. However, here are some general tips on taking chewable tablets: You can try breaking the tablet into smaller pieces before chewing, which may make it easier to chew and swallow. You can also try taking the tablet with a small amount of food, such as yogurt or applesauce, to help with chewing and swallowing. If you continue to have difficulty with chewable tablets, you can talk to your doctor or pharmacist about alternative forms of the medication, such as capsules or liquid. It's important to always follow the instructions provided by your healthcare provider or the medication label when taking any medication. If you have concerns or questions about your medication, don't hesitate to speak with your doctor or pharmacist. | Appropriate/Appropriate | High | Appropriate | -- |
| 62 | medication information | Patients | How to use Loceryl nail lacquer(Amorolfine)? | Loceryl nail lacquer (amorolfine) is a topical antifungal medication used to treat fungal nail infections. Here are the general steps to use Loceryl nail lacquer: Before applying the lacquer, make sure to clean the affected nail(s) with a swab or cotton pad and remover supplied with the product. This is important to ensure that the nail is clean and dry before applying the lacquer. Using the applicator brush supplied, apply a thin layer of Loceryl nail lacquer to the entire surface of the affected nail(s). Make sure to cover the entire nail surface, including the edges and the undersurface of the nail. Allow the lacquer to dry for a few minutes before putting on socks, shoes or other footwear. This will help to ensure that the medication is absorbed into the nail. Repeat the application once a week, using the same applicator brush. It is important to continue using the medication as directed, even if the infection appears to have cleared up, as fungal infections can recur if not treated adequately. If the infection has not improved after several weeks of treatment, or if you experience any side effects, speak to your doctor or pharmacist for advice. | Appropriate/Appropriate | High | Appropriate | -- |
| 63 | medication information | Patients | What is the proper way to keep nitroglycerin sublingual tablets? | As an AI language model, I do not have personal experience, but according to the information provided by the manufacturer, nitroglycerin sublingual tablets should be kept in their original container, tightly closed and protected from light, moisture, and heat. The ideal temperature for storage is between 15-30 degrees Celsius (59-86 degrees Fahrenheit). Nitroglycerin tablets are sensitive to heat and moisture, and exposure to these factors can reduce their effectiveness. The tablets should also be kept out of reach of children and pets. If the tablets become discolored, show signs of deterioration, or are past their expiration date, they should be discarded properly. It is advisable to check with your healthcare provider or pharmacist if you have any questions or concerns regarding the proper storage and use of nitroglycerin sublingual tablets. | Appropriate/Appropriate | High | Appropriate | -- |
| 64 | medication information | Patients | I have been taking Amiodarone for 10 days, and in the last 5 days, I have insomnia, nightmare and wake up in the midnight, wondering if Amiodarone is the cause of it? | As an AI language model, I cannot provide medical advice or diagnose conditions. However, insomnia is a known side effect of Amiodarone. It is recommended that you speak with your healthcare provider if you experience any side effects while taking medication. They will be able to evaluate your symptoms and determine the best course of action. | Inappropriate/Inappropriate | High | Inappropriate | Chatgpt gave an incorrect answer |
| 65 | medication information | Patients | Can pregnant women use Cefixime ? | Cefixime belongs to the third generation cephalosporin antibiotics and caution is advised when used by pregnant women. Although there is no direct evidence showing that Cefixime is harmful to pregnant women or fetuses, it is recommended that pregnant women consult with their doctor before using any medication. The doctor will assess the individual situation of the pregnant woman, weigh the pros and cons, and decide whether to recommend the use of Cefixime or alternative medications. | Appropriate/Appropriate | High | Appropriate | -- |
| 66 | medication information | Patients | I have severe photophobia after using Ivabradine and I am worried about damaging my vision. What should I do? | Ivabradine is a medication used to treat heart conditions such as angina and heart failure. One of the side effects of this medication is visual disturbances, including photophobia or sensitivity to light.  If you are experiencing severe photophobia after taking Ivabradine, it is important to talk to your doctor immediately. They may adjust your medication dosage or recommend an alternative treatment. It is also important to protect your eyes from bright lights by wearing sunglasses or avoiding exposure to bright light whenever possible.  In general, any significant changes in vision should be reported to a healthcare professional as soon as possible, especially if they are associated with medication use. | Appropriate/Appropriate | High | Appropriate | -- |
| 67 | medication information | Patients | Will Isosorbide worsen glaucoma syndrome? | Isosorbide is a vasodilator mainly used to treat cardiovascular diseases and is not directly related to exacerbating symptoms of glaucoma. However, if a patient with glaucoma takes Isosorbide, they should inform their doctor because certain medications may affect glaucoma. The doctor will assess the individual case and choose the most appropriate treatment plan. | Appropriate/Appropriate | High | Appropriate | -- |
| 68 | medication information | Patients | Can Tranexamic Acid be taken during the menstruation period? | Tranexamic Acid can be used to treat excessive or prolonged menstrual bleeding, so it is a common application during menstruation. However, please note that before using Tranexamic Acid, you should read the drug instructions carefully and follow the doctor's advice and prescription. If you have any concerns or discomfort, you should seek medical assistance immediately. | Appropriate/Appropriate | High | Appropriate | -- |
| 69 | medication information | Patients | Is there any possibility of increasing the cancer risk when taking Duodart 0.5mg/0.4mg Cap? | Duodart capsule contains Dutasteride and Tamsulosin as its main components and is used to treat benign prostatic hyperplasia (BPH). There is no evidence to suggest that the use of Duodart is associated with an increased risk of cancer, but patients should closely monitor for any unusual symptoms and promptly report them to their doctor while using the medication. If patients have any concerns or questions, they should consult their doctor. | Appropriate/Inappropriate | Low | Appropriate | -- |
| 70 | medication information | Patients | What is the pregnancy category for XYZAL and ACETAMINOPHEN? | XYZAL (Levocetirizine) is classified as L2 for breastfeeding, which means its safety is unknown and its use is recommended with caution. There is currently insufficient research evidence to determine whether Levocetirizine affects the health of infants, so breastfeeding women are advised to evaluate the risks and benefits before use. Acetaminophen is classified as L1 for breastfeeding, which is the safest drug classification and can be safely used in breastfeeding women. Most studies have shown that Acetaminophen does not have any significant adverse effects on breastfeeding infants. | Appropriate/Appropriate | High | Appropriate | -- |
| 71 | medication information | Patients | Can men try to conceive while taking Hydroxyurea 500mg/cap? | Men using Hydroxyurea should take appropriate contraceptive measures after consulting a doctor to avoid adverse effects on fertility. Hydroxyurea is classified as a category D drug for pregnant women, which means adverse effects on fetal development have been found in animal studies, but human study data is very limited. Therefore, women should tell their doctors whether they are pregnant or planning to become pregnant before using Hydroxyurea and use effective contraceptive measures during its use. The impact of Hydroxyurea on men's use is still unclear, but it is still recommended to take appropriate contraceptive measures during use. It is recommended to make a decision after consulting a doctor. | Appropriate/Appropriate | High | Appropriate | -- |
| 72 | medication information | Patients | Can I take pain relievers before meal? | The timing and whether to take pain relievers before or after meals depend on the type of pain reliever and individual physical condition. Some pain relievers need to be taken before or after meals to reduce their irritating effects on the stomach. However, some pain relievers do not need to be taken at a specific time and can be taken at any time. Therefore, it is best to consult a doctor or pharmacist for advice to determine the best timing and method of use for the pain reliever. | Inappropriate/Inappropriate | High | Inappropriate | ChatGPT remained neutral and did not provide any suggestions |
| 73 | medication information | Patients | Will long-term use of Famotidine 20 mg affect kidney function? | Famotidine is an antihistamine medication used to treat gastrointestinal problems such as acid reflux and peptic ulcers. Long-term use of famotidine may have an impact on kidney function, especially in people who already have kidney problems. Therefore, it is recommended to consult a doctor and undergo relevant kidney function tests before using famotidine long-term to determine if it is safe. If any symptoms of kidney problems occur, they should be reported to the doctor immediately. | Appropriate/Appropriate | High | Appropriate | -- |
| 74 | medication information | Patients | What is the use of clopidogrel? | Clopidogrel is an antiplatelet medication used to prevent cardiovascular events such as heart attacks, stroke, and peripheral artery disease. It can inhibit platelet aggregation, reduce the risk of thrombosis, and thus reduce the occurrence of cardiovascular events. | Appropriate/Appropriate | High | Appropriate | -- |
| 75 | medication information | Patients | What is the timing to use antipyretic syrup? Is it a must to use after meal? | Usually, antipyretic syrups are recommended for use during a fever and should be used according to the medication's instructions. Most antipyretic syrups do not have an absolute requirement for before or after meals, but the timing of use can be selected based on the characteristics of the medication. For example, some antipyretic medications may irritate the stomach, so they can be used after meals to alleviate discomfort. If you have any questions about medication use, it is recommended to consult a doctor or pharmacist for professional advice. | Appropriate/Appropriate | High | Appropriate | -- |
| 76 | medication information | Patients | What should I do when large red rashes appear after taking tamoxifen? | The appearance of a large rash on the skin may be one of the side effects of tamoxifen. It is recommended to report this symptom to the doctor as soon as possible. The doctor may recommend discontinuing tamoxifen until the symptoms disappear or are alleviated. If the symptoms are severe, the doctor may recommend other treatment methods. In addition, patients should avoid using any new medications or cosmetics to avoid further irritation to the skin. | Appropriate/Appropriate | High | Appropriate | -- |
| 77 | medication information | Patients | How Febuxostat. F.C. 80mg metabolize in our body? | Febuxostat is metabolized primarily by the liver, with the involvement of the CYP450 enzyme system. It undergoes both oxidation and glucuronidation, with the major metabolites being the 5'-hydroxyfebuxostat and the corresponding acylglucuronide. These metabolites are then eliminated primarily through the renal route. | Appropriate/Appropriate | High | Appropriate | -- |
| 78 | medication information | Patients | The patient was having headaches after taking LEVOFLOXACIN 500mg/Tab, is there any possibility that LEVOFLOXACIN is the cause of it? | Headaches after taking Levofloxacin may be related to the medication, but they may also be caused by other factors. It is recommended to ask the patient if they have any other discomfort symptoms, such as nausea, vomiting, dizziness, etc. If the symptoms persist or worsen, it is recommended to consult a doctor immediately. | Appropriate/Appropriate | High | Appropriate | -- |
| 79 | medication information | Patients | I need to know the details about STATIN-related rhabdomyolysis. | Rhabdomyolysis is a muscle disease in which the muscle fibers in muscle cells dissolve, causing muscle protein to be released into the blood. Rhabdomyolysis can cause symptoms such as muscle pain, muscle weakness, and muscle soreness. In cases where this condition occurs, there are corresponding biomarkers in the patient's blood, such as creatine kinase (CK). Some studies suggest that STATIN medications may increase a patient's risk of developing rhabdomyolysis. Patient risk factors include high-dose use, renal insufficiency, thyroid dysfunction, family history, age, etc. If a patient experiences symptoms such as muscle pain, muscle weakness, or muscle soreness, it is recommended to consult a doctor immediately. | Appropriate/Appropriate | High | Appropriate | -- |
| 80 | medication information | Patients | Is it normal for a child to have diarrhea (1-2 times/day) while taking cefixime? | Diarrhea is a common side effect when using antibiotics, but the severity of diarrhea can vary from person to person. If the diarrhea is not severe and the child has a good appetite and fluid intake, and there are no other symptoms, it may not require special treatment, but it is still recommended to inform the doctor for better advice. If the diarrhea is severe, the child has no appetite, loses fluids, experiences abdominal pain, fever, bloody stools, and other symptoms, immediate medical attention should be sought. | Appropriate/Appropriate | High | Appropriate | -- |

^a^ All the questions we tested and the answers generated by ChatGPT were in Chinese. The supplemental tables were eventually translated into English for the final version.

^b^ The annotations were used to determine the appropriateness of the AI-generated responses. The discordance rate between the pharmacists' annotations was calculated and resolved by a final annotation.

**eTable 2. ChatGPT responses for Drug-Herb Interactions questions and three pharmacists' annotations**

| **No.** | **Questions ^c^** | **Answer by ChatGPT ^c^** | **Assesment by pharmacist A/B** | **Confidence** | **Assesment by pharmacist C ^d^** | **The cause of Inappropriate** | **Reference** |
| --- | --- | --- | --- | --- | --- | --- | --- |
| 1 | Can aspirin be taken with ginseng? | The concurrent use of Panax ginseng (ginseng) and aspirin may increase the risk of bleeding because ginseng may affect platelet function and coagulation mechanisms. If simultaneous use of ginseng and aspirin is necessary, it is recommended to consult a physician or pharmacist beforehand to determine the safe dosage and timing of medication. Regular monitoring of blood coagulation function should also be conducted to ensure overall health. | Appropriate/Appropriate | High | Appropriate | -- | Concurrent use of herbal products with anticoagulant/antiplatelet effects, such as ginseng, alongside other herbs or drugs with similar properties could heighten bleeding risk. Consequently, careful monitoring for signs and symptoms of bleeding is recommended when these agents are used together. (UpToDate, Risk Level C, Moderate, Fair) |
| 2 | Can aspirin be taken with saffron? | Saffron is a common herb that is sometimes used to treat certain conditions such as pain, inflammation, and depression. However, there is a lack of sufficient research evidence regarding the safety and efficacy of using saffron together with aspirin, so it cannot be determined whether they can be safely used together.  In addition, aspirin itself is a medication that has similar anti-inflammatory and pain-relieving effects, so it is recommended that you do not use other similar herbs or supplements while using aspirin to avoid potential interactions or unnecessary risks.  If you plan to use saffron or other herbs to treat a certain condition, it is recommended that you consult with your doctor or pharmacist first to determine their safety and efficacy. If you are using aspirin to treat a certain condition, you should also inform your doctor or pharmacist to ensure the safe use of the medication. | Appropriate/Inappropriate | Low | Inappropriate | ChatGPT gave an incorrect answer | While the UpToDate database doesn't indicate a drug-drug interaction (DDI) between saffron and aspirin, traditional Chinese medicine posits that crocin in saffron exerts an antiplatelet aggregation effect. Hence, concurrent usage is preferably avoided. If coadministration is necessary, vigilant monitoring for bleeding is advised, and immediate medical attention should be sought if necessary.. |
| 3 | Can aspirin be taken with licorice (Glycyrrhiza glabra)? | It is generally not recommended to take aspirin and licorice (Glycyrrhiza glabra) together without consulting a healthcare provider first. Licorice contains compounds that can increase the risk of bleeding, and when combined with aspirin, which is also a blood thinner, it can increase the risk of bleeding even further.  Additionally, licorice can also affect how the body processes aspirin, potentially increasing its side effects. Therefore, it is important to consult with a healthcare provider before taking aspirin and licorice together to ensure it is safe and appropriate for your individual health needs. | Appropriate/Inappropriate | Low | Appropriate | -- | Micromedex categorizes the risk as moderate and fair concerning the concurrent use of Licorice and antiplatelet agents, which may increase the bleeding risk. Theoretically, licorice may enhance the effects of antiplatelet agents. It is suggested that licorice root contains coumarins or coumarin derivatives (Heck et al, 2000). A specific 2-arylcoumarin derivative of licorice, GU-7, exhibited antiplatelet activity in vitro, potentially through an increase in intraplatelet cyclic AMP (Norred & Brinker, 2001; Newall et al, 1996). Glycyrrhizin, a component of licorice, showed selective thrombin inhibition in vitro (Francischetti et al, 1997). Therefore, caution is advised. |
| 4 | Can aspirin taken with Astragalus membranaceus? | Aspirin and Astragalus membranaceus are two different substances with different properties and effects, so it is important to use caution when combining them.  Aspirin is a nonsteroidal anti-inflammatory drug (NSAID) commonly used to relieve pain, reduce fever, and prevent blood clots. It works by inhibiting the production of prostaglandins, which are chemicals that promote inflammation, pain, and fever.  Astragalus membranaceus, on the other hand, is a traditional Chinese herb that has been used for centuries to enhance the immune system, protect the liver, and reduce inflammation. It contains a variety of compounds, including flavonoids, saponins, and polysaccharides, that are believed to be responsible for its health benefits.  While there is no known interaction between aspirin and Astragalus membranaceus, it is always important to consult with a healthcare provider before taking any new supplements or medications, especially if you have any underlying medical conditions or are taking other medications.  Some potential concerns with combining aspirin and Astragalus membranaceus may include an increased risk of bleeding due to the antiplatelet effects of aspirin and the potential blood-thinning effects of Astragalus membranaceus. Additionally, Astragalus membranaceus may interact with certain medications, including immunosuppressants and anticoagulants, so it is important to discuss any potential interactions with a healthcare provider before taking this herb. | Inappropriate/Inappropriate | High | Inappropriate | ChatGPT remained neutral and did not provide any suggestions | According to UpToDate, no interactions of Risk Level A or greater have been identified.  Micromedex posits a moderate, fair risk where concurrent use of Astragalus and antiplatelet agents might lead to an increased bleeding risk. Astragalus may heighten fibrinolysis, inhibit thromboxane A2 synthesis, and augment prostaglandin I2. Therefore, consider discontinuing Astragalus preoperatively to mitigate the risk of surgical bleeding complications.  For patient education, it is advised to maintain a minimum interval of 2 hours between consuming Chinese and Western medicine. |
| 5 | Can asprin be taken with Coptis chinensis? | There is limited information available on the potential interactions between aspirin and Coptis chinensis, a traditional Chinese herbal medicine. It is important to consult with a healthcare professional or a qualified herbalist before combining any medications or supplements, including aspirin and Coptis chinensis.  Coptis chinensis is known to contain berberine, which may affect the way some medications, including aspirin, are processed by the body. Some studies have suggested that berberine may enhance the effects of aspirin, while others have suggested that it may reduce the effectiveness of aspirin or increase the risk of bleeding.  Therefore, it is important to consult with a healthcare professional before combining aspirin and Coptis chinensis, especially if you have any medical conditions or are taking any other medications or supplements. Your healthcare professional can advise you on the appropriate dosages and potential risks associated with combining these substances. | Inappropriate/Inappropriate | High | Inappropriate | ChatGPT response is not detailed enough | According to Micromedex and UpToDate, no interactions have been identified. During patient education, individuals are reminded to maintain at least a 2-hour interval between the intake of Chinese and Western medicine. |
| 6 | Can aspirin be taken with hawthorn? | It is generally safe to take aspirin and hawthorn together, but it is important to talk to a healthcare professional before combining any medications or supplements. Aspirin is a nonsteroidal anti-inflammatory drug (NSAID) that is commonly used to reduce pain, fever, and inflammation. Hawthorn is an herbal supplement that is often used to support heart health and improve circulation. Although hawthorn is generally considered safe, it may interact with certain medications, including those that affect blood pressure or blood thinning. Aspirin is a blood-thinning medication, so taking it with hawthorn may increase the risk of bleeding. Therefore, it is important to talk to a healthcare professional before taking aspirin and hawthorn together, especially if you have a bleeding disorder or are taking other medications that may interact with hawthorn. Your healthcare professional can advise you on the appropriate dose and timing of these medications and supplements to minimize the risk of side effects or interactions. | Inappropriate/Inappropriate | High | Inappropriate | ChatGPT response is not detailed enough | According to UpToDate, no interactions of Risk Level A or greater have been identified. Micromedex, however, suggests a moderate, fair risk associated with the concurrent use of Hawthorn and antiplatelet agents (including aspirin), which may lead to an increased risk of bleeding. Theoretically, several active components of Hawthorn extract might elevate the bleeding risk if consumed with antiplatelet agents. These active molecules, specifically vitexin-2-o-rhamnoside (V2R) and hyperoside, inhibit the biosynthesis of thromboxane A2, a primary mediator of platelet aggregation (Vibes et al, 1994). |
| 7 | Can aspirin be taken with Angelica sinensis? | It is always best to consult with a healthcare professional before taking any new medication or herbal supplement, especially if you are currently taking any other medications or have a medical condition.  That being said, there are some potential interactions between aspirin and Angelica sinensis, also known as dong quai, which is an herb commonly used in traditional Chinese medicine. Both aspirin and dong quai can thin the blood and increase the risk of bleeding, so taking them together could potentially increase the risk of bleeding.  If you are considering taking both aspirin and Angelica sinensis, it is important to discuss the potential risks and benefits with your healthcare provider to determine if it is safe and appropriate for you. Your healthcare provider may recommend monitoring your blood clotting levels if you take these two substances together. | Appropriate/Inappropriate | Low | Appropriate | -- | While Micromedex and UpToDate indicate no interaction between aspirin and Angelica, the Journal of Taiwan Pharmacy discusses the potential effects of combining traditional Chinese and Western medicines. Danggui, a Chinese medicine known for promoting blood circulation and removing blood stasis, exhibits inhibitory effects on platelet aggregation. Therefore, when coadministered with Western medicines possessing anticoagulant effects, such as aspirin, it could potentially heighten the risk of bleeding. Consequently, concurrent use is not recommended. |
| 8 | Can aspirin be taken with ginkgo? | It is generally safe to take aspirin and ginkgo biloba together, but it's always a good idea to check with a doctor or pharmacist before taking any new medication or supplement. Both aspirin and ginkgo can affect blood clotting, so it's important to be cautious if you're taking other medications that also affect blood clotting, such as warfarin or heparin. Ginkgo can also increase the risk of bleeding, especially when taken in high doses, so it's important to use caution and follow recommended dosages. Overall, it's best to consult with a healthcare professional before taking any new medication or supplement, especially if you have any underlying health conditions or are taking other medications. | Appropriate/Appropriate | High | Appropriate | -- | According to DynaMed, the concurrent use of Ginkgo and nonsteroidal anti-inflammatory agents (NSAIDs) may elevate the risk of bleeding. Clinical management suggests avoiding coadministration of Ginkgo and NSAIDs. If these agents are used together, it's recommended to regularly monitor the patient for bleeding time and signs and symptoms of excessive bleeding to assess if Ginkgo has adversely affected platelet function. |

^c^ All the questions we tested and the answers generated by ChatGPT were in Chinese. The supplemental tables were eventually translated into English for the final version.

^d^ The annotations were used to determine the appropriateness of the AI-generated responses. The discordance rate between the pharmacists' annotations was calculated and resolved by a final annotation.
